# Cyber Security: Effects of Penalizing Defenders in Cyber-Security Games via Experimentation and Computational Modeling

**DOI:** 10.3389/fpsyg.2020.00011

**Published:** 2020-01-28

**Authors:** Zahid Maqbool, Palvi Aggarwal, V. S. Chandrasekhar Pammi, Varun Dutt

**Affiliations:** ^1^Applied Cognitive Science Laboratory, Indian Institute of Technology Mandi, Kamand, India; ^2^Dynamic Decision Making Laboratory, Carnegie Mellon University, Pittsburgh, PA, United States; ^3^Centre of Behavioural and Cognitive Sciences, University of Allahabad, Allahabad, India

**Keywords:** monetary penalties, defenders, adversaries, cybersecurity, decision-making, instance-based learning theory, recency, frequency

## Abstract

Cyber-attacks are deliberate attempts by adversaries to illegally access online information of other individuals or organizations. There are likely to be severe monetary consequences for organizations and its workers who face cyber-attacks. However, currently, little is known on how monetary consequences of cyber-attacks may influence the decision-making of defenders and adversaries. In this research, using a cyber-security game, we evaluate the influence of monetary penalties on decisions made by people performing in the roles of human defenders and adversaries via experimentation and computational modeling. In a laboratory experiment, participants were randomly assigned to the role of “hackers” (adversaries) or “analysts” (defenders) in a laboratory experiment across three between-subject conditions: Equal payoffs (EQP), penalizing defenders for false alarms (PDF) and penalizing defenders for misses (PDM). The PDF and PDM conditions were 10-times costlier for defender participants compared to the EQP condition, which served as a baseline. Results revealed an increase (decrease) and decrease (increase) in attack (defend) actions in the PDF and PDM conditions, respectively. Also, both attack-and-defend decisions deviated from Nash equilibriums. To understand the reasons for our results, we calibrated a model based on Instance-Based Learning Theory (IBLT) theory to the attack-and-defend decisions collected in the experiment. The model’s parameters revealed an excessive reliance on recency, frequency, and variability mechanisms by both defenders and adversaries. We discuss the implications of our results to different cyber-attack situations where defenders are penalized for their misses and false-alarms.

## Key Points

• Penalizing security defenders could be effective ways for organizations to improve their productivity as well as performance.
• We perform a laboratory experiment involving participants to evaluate how penalizing defenders influence attack-and-defend actions in simulated cyber-security games.
• Penalizing defenders for misses causes them to increase the defend proportions; whereas, penalizing them for false-alarms causes them to decrease the defend proportions. Attack proportions increased as a result of less no of defends by the defender in Penalizing defenders for false alarms and decreased in case of penalties for misses.
• Based upon the experiment, penalizing defenders for misses and false-alarms may cause both adversaries and defenders roles to deviate from their Nash proportions.
• A model based upon Instance-based Learning Theory could account for adversary and defender decisions in both conditions where defenders are penalized and where adversaries are awarded.

## Introduction

Cyber-attacks are increasing, and these attacks cause widespread socio-economic damages to society and governance (Forbes, 2016; ThreatMetrix, 2016). Adversaries, people who break into computer systems using bugs and exploits, wage cyber-attacks that cause major disruptions in society (Webroot, 2015). For example, in 2016, a major cyber-attack was carried out against the power-grid in Ukraine (DARKReading, 2016). The power-grid went down, leaving about 50% of homes with a population of 1.4 million without power for several hours (DARKReading, 2016).

Given the widespread damages due to cyber-attacks, organizations have engaged defenders, people who defend and protect computer networks from cyber-attacks (Dutt et al., 2013; Truity, 2015; Maqbool et al., 2017; Prospects, 2017). Defenders may miss detecting an attack on computer systems, or they may commit false-alarms (Dutt et al., 2013; Han and Dongre, 2014; Maqbool et al., 2017; Shang, 2018a). In both situations, it is likely that defenders and their organizations may face reputation damage and monetary consequences (CSIS, 2014; CSO, 2016; Ponemon, 2017; The Guardian, 2017), where some of these outcomes may even entail layoffs (CSO, 2017). For example, as per a recent survey, a technology investment that led to a data or security breach (i.e., missing to detect an attack) was considered a “fireable offense” by 38 to 39 percent of organizations (CSO, 2017). Similarly, committing of false-alarms by defenders may result in short-term or prolonged loss of availability, which can paralyze a company with higher costs, lost revenue, and reputational damage (Gheyas and Abdullah, 2016). Overall, it is expected that such monetary consequences are likely to influence defender’s decisions.

Furthermore, it is likely that the knowledge of defenders’ misses and false-alarms may indirectly cause adversary to change their attack patterns (Dutt et al., 2013; Gheyas and Abdullah, 2016; Maqbool et al., 2017). For example, if defenders are penalized for false-alarms, then they are likely to reduce their defending actions. In such situations, adversary may indirectly attack the network more due to the reduction in defending actions. Similarly, if defenders are penalized for misses, then they are likely to increase their defending actions. Again, adversary may indirectly attack the network less due to the increase in defending actions.

The primary goal of this paper is to understand how bounded rational adversaries and defenders would be affected by punishments for their errors in cybersecurity tasks. Also, an additional goal is to develop cognitive models of adversary’s and defender’s decisions to understand their cognitive processes and to test how these cognitive models perform compared to other rational models. Specifically, this paper studies how penalizing defenders for false alarms and misses influences the decisions of both defenders and adversaries in simulated cyber-security games. We compare the performance of defenders and adversaries to the rational Nash strategies, where the Nash strategies provide the best decision a player can take given the other player’s decisions. This comparison helps explain how penalizing defenders makes defenders and adversaries deviate from optimal Nash strategies. Furthermore, we develop models of cognition (Busemeyer and Diedrich, 2009; Dutt et al., 2013) for the adversary’s and defender’s decisions, and these models help explain the reasons for the deviation of players’ actions from their Nash strategies on account of cognitive limitations of memory and recall.

A way of studying the influence of monetary penalties on the interaction between defenders and adversaries is via behavioral game theory (Camerer, 2003; Alpcan and Basar, 2010; Maqbool et al., 2017). For example, Maqbool et al. (2017) studied this interaction using cyber-security games. In these games, higher rewards for waging successful attacks or detecting attacks caused adversaries and defenders to increase their attack and defend actions, respectively (Maqbool et al., 2017).

In the literature on cognitive theories of decision-making (Dutt et al., 2013), Instance-based Learning Theory (IBLT) (Lebiere, 1999; Gonzalez and Dutt, 2011; Kaur and Dutt, 2013; Maqbool et al., 2016), a theory of decisions from experience in dynamic scenarios, has been used to explain decisions made by participants performing as defenders and adversaries. Maqbool et al. (2017) used IBLT to explain their experimental result. However, Maqbool et al. (2017) did not test the effect of monetary penalties on defender’s decisions. Furthermore, these authors made human players play against their Nash opponents and not against human opponents, where human strategies are likely to be adaptive compared to Nash strategies (Dutt et al., 2013). Also, although Maqbool et al. (2017) explained their results based upon IBLT, they did not test the ability of computational models based upon IBLT to account for human decisions. Overall, the main contribution of this paper is to overcome these limitations in literature.

In this paper, we investigate how monetary penalties on defenders impact the decision-making of defenders and indirectly the decision-making of adversaries in cyber-security games. Also, using IBLT, we suggest specific cognitive processes that influence decisions of adversaries and defenders in cyber-security games. The main novelty of this work is the use of behavioral game theory and IBLT to understand the decisions of adversaries and defenders in scenarios involving punishments to defenders for their errors. To the best of authors’ knowledge, this work is the first of its kind where behavioral game theory and IBLT have been used to understand the role of monetary punishments on decisions of adversaries and defenders.

In what follows, first, we discuss a cyber-security game that we used in this paper. Next, we state our expectations concerning monetary penalties and test these expectations via an experiment and a cognitive model based upon IBLT.

## The Cyber-Security Game

**FIGURE 1:**
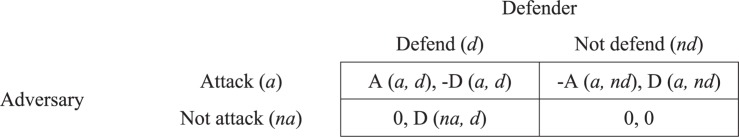
Set of actions and costs for adversaries and defenders. The first value in each cell corresponds to adversary’s cost [e.g., A(a, d)] and the second value corresponds to defender’s cost [e.g., D(a, d)]. Negative cost is a benefit.

Figure 1 shows a cyber-security game (Alpcan and Basar, 2010). In this game, the action set of the adversary includes the attack (*a*) and not-attack (*na*) actions. The action set of the defender includes the defend (*d*) and not-defend (*nd*) actions. The A and D represent costs for attacking and defending, and in this scheme, a negative (−) cost is a benefit. While playing the game, there will be benefits for the adversary [-A(a, nd)] due to successful attacks and costs [A(a, d)] due to unsuccessful attacks. Similarly, there will be benefits for the defender [-D(a, d)] for catching cyber-attacks; and, costs resulting from mounting defense when there is no attack [D(na, d)] and not mounting defense when there is an attack [D(a, nd)]. We use this game-theoretic framework for investigating the role of monetary penalties on attack-and-defend decisions.

## Effect of Monetary Penalties in the Cyber-Security Game

**FIGURE 2:**
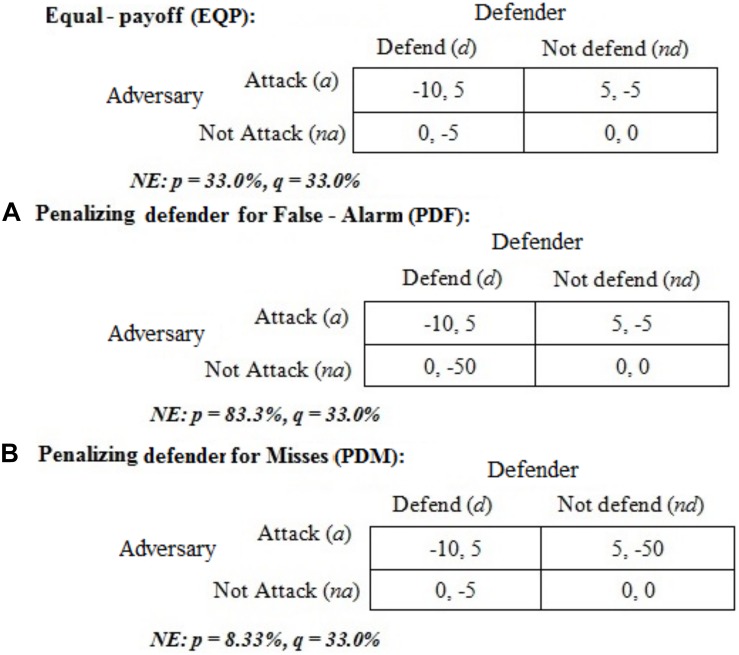
Payoffs for both defenders and adversaries across different conditions. **(A)** Penalizing Defenders for False-alarms (PDF). **(B)** Penalizing Defenders for Misses (PDM). The Nash Proportions of attack *(p)* and defend *(q)* actions are also shown.

We varied the defender’s payoff across different conditions in the cyber-security game (Figure 1) and kept the payoff for the adversary constant. These conditions included, equal payoffs (EQP), penalizing defender for false-alarms (PDF), and penalizing defender for misses (PDM) (see Figure 2).

The EQP condition and its payoffs was proposed by Alpcan and Basar (2010) and it was used by Maqbool et al. (2017). In EQP condition, defenders receive equal penalties (−5) for *a-nd* and *na-d* actions, and this condition acts as a baseline (control) for the PDF and PDM conditions. In PDF condition, defenders are penalized −50 points for a *na-d* action profile, which is 10-times the penalty in EQP. In PDM condition, defenders are penalized −50 points for an *a-nd* action combination, which is 10-times the penalty in EQP condition. Thus, in the PDF condition, defenders are penalized for false-alarms; whereas, in the PDM condition, defenders are penalized for misses. The optimal mixed-strategy Nash proportions for attack actions (*p*) and defend actions (*q*) are also shown in Figure 2 for each condition.

Instance-Based Learning Theory (Gonzalez et al., 2003; Gonzalez and Dutt, 2012) may be applicable for decisions of human adversaries and defenders (Arora and Dutt, 2013; Dutt et al., 2013; Maqbool et al., 2017). According to IBLT, both adversaries and defenders possess cognitive limitations on memory and rely on recency, frequency, and variability mechanisms to make decisions. In the presence of such limitations, human players would tend to take those actions that they perceive as maximizing their expected rewards based upon recent and frequent experiences from their memory (Dutt et al., 2013).

The defenders are penalized −50 points for a *na-d* action combination in the PDF condition. Thus, defenders perceiving losses due to false-alarms would tend to reduce their defending actions in PDF condition compared to EQP condition. The reduction in defending actions would likely result in higher attack proportions in the PDF condition compared to EQP condition as successful attacks give +5 points to the adversaries.

Similarly, in PDM condition, the defender is penalized −50 points for an *a-nd* action combination. Thus, defenders would tend to increase defending actions in PDM condition compared to EQP condition to increase their perceived gains. The increase in the defending proportion in PDM condition would likely result in lower attack proportions by adversaries compared to those in the EQP condition. That is because adversaries will likely get caught and lose −10 points when defenders defend excessively in the PDM condition. As per IBLT, adversaries would tend to minimize this monetary loss by reducing their attack proportions.

## Materials and Methods

### Experimental Design

We evaluated the effects of monetary penalties on attack-and-defend decisions by varying them across three between-subject conditions: EQP (*N* = 50 participants), PDF (*N* = 50 participants), and PDM (*N* = 50 participants). In each condition, human players acting as adversaries or defenders were randomly paired to play against each other in a cyber-security game across 50-repeated trials. For example, in EQP, 25 defenders were randomly paired with against 25 adversaries in the cyber-security game (making a total of 25 participants). In each condition, dependent measures included attack proportions and defend proportions averaged across participants and trials. For computing the attack or defend proportions, each attack/defend action by a participant in a trial was coded as 1 and each not-attack/not-defend action by a participant in a trial was coded as 0. Later, the 1 s and 0 s were averaged across participants and trials to yield different proportions. We used one-way analysis of variance (ANOVA; Field, 2013) to compare performance across different conditions. The alpha level (the probability of rejecting the null hypothesis when it was true) was set at 0.05 and power (the probability of rejecting the null hypothesis when it was false) was set at 0.80.

### Stimulus and Apparatus

**FIGURE 3:**
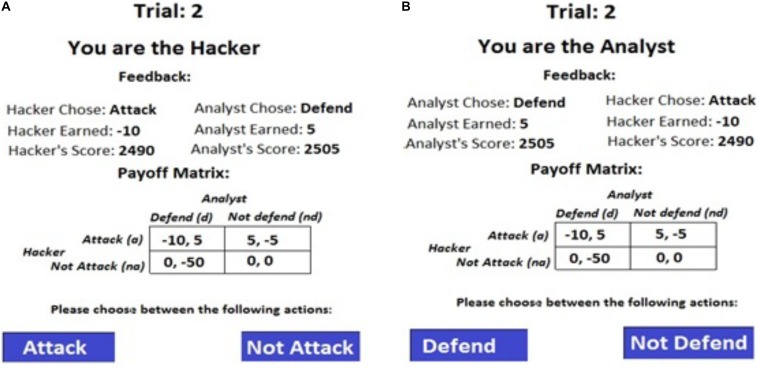
The experimental interface presented to participants in cyber-security game. The interface provided feedback on the actions taken and payoffs obtained in the last trial by both players. Also, the interface showed the cost matrices to participants. The interface seen by participants acting as hackers **(A)** and analysts **(B)** in a trial.

Figure 3 shows the interface shown to participants acting in the roles of “hacker” (adversary) (A) and “analysts” (defender) (B). Participants were given the following feedback from the last trial: payoff matrices, actions chosen by them and their opponents, current payoffs obtained by them and their opponents, and total payoffs obtained by them and their opponents since the start of the experiment. Both participants were asked to choose between attack or non-attack actions (adversary) and defend or not-defend actions (defender) in each trial.

### Participants

This study was carried out in accordance with the recommendations of the Ethics Committee at the Indian Institute of Technology Mandi (IITM/DST-ICPSCPS/VD/251) with written informed consent from all participants. Participation was voluntary and all participants gave written informed consent before starting their study. All participants were students at the Indian Institute of Technology Mandi. Participants were recruited through an online advertisement and participation was voluntary. One hundred sixty-two participants in the age group of 18–30 years (Average = 22.1 years and standard deviation = 2 years) participated in this experiment. Twenty-one percent were females. Participants were from different education levels: 74% were pursuing undergraduate degrees, 22% were pursuing master’s degrees, and 4% were pursuing Ph.D. degrees. Furthermore, all participants were pursuing degrees in engineering disciplines. About 35% of the participants had computer science and engineering degrees, where these participants had done a course in computer networks.

### Procedure

Participants were randomly assigned to different conditions and to the analyst and hacker roles in each condition. Next, instructions regarding the game play, game objectives, and payment were provided to participants. Questions concerning game play, game objectives, and payment were answered before participants could start performing in the game. The experiment started with an initial score of 2500 for both adversaries and defenders participants. This initial score ensured that the total score would not become negative for any player by the end of the game. The experiment took 30-min to complete for a pair of participants playing against each other. Participants received a base payment of 50 cents after they finished their study. Participants were also rewarded based on performance in the game. For calculating the performance incentive, final score in the game was converted to real money in the following ratio: 250 points in the game = 1.5 cents in real money. The maximum and minimum performance bonus possible across different conditions was 11 cents and 0 cents, respectively.

## Results

### Proportion of Attack-and-Defend Actions

**FIGURE 4:**
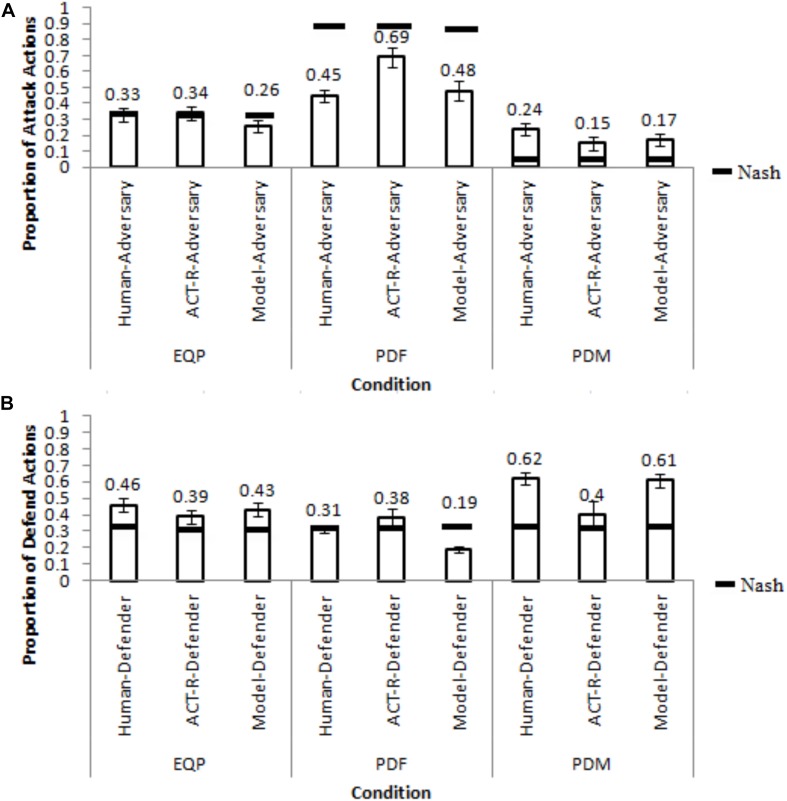
Attack and defend proportions across different conditions from human participants, ACT-R model and the IBL model. **(A)** The comparison between model and human adversaries. **(B)** The comparison between model and human defenders. The black line on each bar shows the corresponding Nash proportions and the error bars represent the 95% confidence interval.

One-way ANOVAs were performed on attack-and-defend proportions with conditions as a between-subjects factor. Figures 4A,B show the attack-and-defend proportions in different conditions from human participants and the corresponding Nash equilibria (the model results will be discussed ahead in the paper).

As seen in Figures 4A,B, the attack-and-defend proportions were influenced by the condition (attack: *F*(2, 72) = 12.881, *p* < 0.05, *w*^2^ = 0.24; defend: *F*(2, 72) = 10.839, *p* < 0.05, *w*^2^ = 0.21; where, *w*^2^ is the effect-size). The Student-Newman-Keuls (SNK; Field, 2013) *post hoc* test revealed that the attack proportions were significantly greater in the PDF condition (0.45) compared to in the EQP condition (0.33) (*p* < 0.05). Similarly, the attack proportions were significantly smaller in the PDM condition (0.24) compared to in the EQP condition (0.33) (*p* < 0.05). Furthermore, the defend proportions were significantly smaller in the PDF condition (0.31) compared to in the EQP condition (0.46) (*p* < 0.05). Also, the defend proportion in the PDM condition were significantly higher than those in the EQP condition (0.62 > 0.46; *p* < 0.05). Overall, these results are as per our expectations.

The attack proportions did not deviate from the Nash level in EQP condition (0.33 ∼ 0.33; *t*(24) = 0.042, *p* = 0.97, *r* = 0.008, where *r* is the effect-size). However, the attack proportions deviated significantly from their Nash levels in the PDF and PDM conditions (PDF: 0.45 < 0.83; *t*(24) = −10.413, *p* < 0.001, *r* = 0.90; PDM: 0.24 > 0.08; *t*(24) = 5.404, *p* < 0.001, *r* = 0.73). The defend proportions deviated significantly from their Nash levels in the EQP condition (0.46 > 0.33, *t*(24) = 3.307, *p* < 0.05, *r* = 0.54) and in the PDM condition (0.62 > 0.33; *t*(24) = 5.379, *p* < 0.001, *r* = 0.73). However, defend proportions did not deviate from their Nash level in the PDF condition (0.31 ∼ 0.33; *t*(24) = −0.994, *p* = 0.30, *r* = 0.33). Overall, in a majority of conditions there was a significant deviation from the Nash levels for both adversary and defenders.

## The IBL Model

The cognitive model of adversary and defender is implemented using IBLT (Gonzalez et al., 2003). IBLT has been used to model decision making processes of adversaries, defenders and end-users in various cybersecurity situations (Aggarwal et al., 2017, 2018; Cranford et al., 2019a, b).

We developed a model based upon IBLT to explain our experimental results. An instance, smallest unit of experience, in the IBL model consists of three parts: a situation in a task (a set of attributes that define the decision situation), a decision of choosing an alternative in a task, and an outcome resulting from choosing an alternative in that situation. In the IBL model, instances accumulate over time, are retrieved from memory according to the activation strength in memory. This activation strength of instances is measured by a statistical mechanism called activation, originally implemented in the ACT-R architecture (Anderson and Lebiere, 1998). The activation relies on the frequency and recency of experienced choices and outcomes. IBLT assumes that the instances experienced by the decision maker are activated in memory as a function of their previous occurrence: more recent and frequent instances are more active in memory than less recent and less frequent ones. We make two single-person IBL models (one for the adversary and the other for the defender) to interact with each other in the game shown in Figure 1 repeatedly.

In the IBL model, each instance corresponds to an alternative to choose for a player (i.e., to *attack* or *not-attack* for the adversary and to *defend* or *not-defend* for the defender) and the outcome obtained (e.g., −10 points for the adversary for a caught attack). As the situation remains the same for each binary decision, the structure of an instance is simply [alternative, outcome]. In each trial *t* of the game, the process of choosing an alternative in the model for a player starts with calculation of blended values for each alternative based on the previously observed outcomes in similar situations. The blended value of alternative j is defined as:

(1) $${\text{v}}_{\text{j}}=\sum_{\text{i}=1}^{\text{n}}{\text{p}}_{\text{ij}}\,{\text{x}}_{\text{ij}}$$

Where, p_ij_ is the probability of retrieval of instance *i* corresponding to alternative *j* from memory; x_ij_ is the outcome stored in instance *i* corresponding to alternative *j*; and, *n* is the total number of instances corresponding to the alternative *j* in memory. The alternative with the highest blended value is selected by the model in each trial (Dutt et al., 2013). The equation 1 defines that the blended value for each option is the sum of all observed outcomes weighed by their probability of retrieval. The probability of retrieval is a function of activation which is defined as:

(2) $${\text{p}}_{\text{ij}}=\frac{{\text{e}}^{\frac{{\text{A}}_{\text{ij}}}{\tau }}}{\sum_{i=1}^{\text{n}}{\text{e}}^{\frac{{\text{A}}_{\text{ij}}}{\tau }}}$$

Where, A_ij_ is the activation of instance *i* corresponding to alternative *j* in memory; τ is random noise defined as $\tau =\sigma \times \sqrt{2}$; and, σ is a free parameter called noise to capture the imprecision of recalling past experiences from memory (details below). The activation of each instance in memory depends upon a mechanism from ACT-R (Anderson and Lebiere, 1998). The activation of an instance in a given trial is a function of the frequency of its outcome’s occurrence and the time difference between the current time and past times when the instance’s outcome occurred in the task. At each trial t, the activation A_i_ of an instance *i* is defined as:

(3) $${\text{A}}_{\text{i}}=\ln\left(\sum_{{\text{t}}_{p,i}\in \left\{1,\ldots ,\text{t}-1\right\}}{\left(\text{t}-{\text{t}}_{p,i}\right)}^{-\text{d}}\right)+\sigma \cdot \ln\left(\frac{1-\gamma_{\text{i},\text{t}}}{\gamma_{\text{i},\text{t}}}\right)$$

Where, *d* and *σ* are free parameters called memory decay and cognitive noise; *t* is the current trial; t_p,i_ are the previous trials where the instance *i* with an outcome was created or the instance’s activation was reinforced due to outcome’s occurrence in the task; and, γ_*i,t*_ is a random draw from a uniform distribution in trial *t* that is bounded between 0 and 1. The summation in the first term in equation 3 includes the frequency of observations and the difference of two time periods correspond to the recency of observations.

Therefore, the activation of an instance corresponding to an observed outcome increases with the frequency of observation of outcomes in the task (i.e., by increasing the number of occurrences in the summation) and with the recency of those outcome observations (i.e., by t−t_p,i_ differences that correspond to that instance in memory). The decay parameter *d* has a default value of 0.5 in ACT-R and it captures the rate of forgetting. The higher the value of the *d* parameter, the more is the reliance on recency and the faster is the decay of memory. The $\sigma \cdot \ln\left(\frac{1-\gamma_{\text{i},\text{t}}}{\gamma_{\text{i},\text{t}}}\right)$ term represents Gaussian noise, which represents the random error to the activation process and it is intended to represent the noise associated with memory activation. The higher the σ value, the more variability there will be in instance activations and in trial-to-trial decisions. As per IBLT, we expect a decrease in the defend proportions when the defender is penalized for false alarms and an increase in defend proportions when the defender is penalized for misses. Furthermore, we expect an increase in the attack proportions when the defender is penalized for false alarms and a decrease in attack proportions when the defender is penalized misses.

### Calibration of Model Parameters

We assumed two versions of the IBL models. One version of the IBL model used calibrated values of *d* and σ parameters (referred to as “calibrated model”) and the other version of the IBL model used the ACT-R default values for the *d* and σ parameters (referred to as “ACT-R model”). The ACT-R model refers to an agent that relies less upon recency, frequency, and variability in decisions. In the calibrated model, the *d* and σ parameters for the adversary were equated with *d* and σ parameters of the defender, respectively. Thus, both adversary and defender agents possessed the same values of the calibrated *d* and σ parameters in the calibrated model. The reason for equating the parameters of adversary and defender agents was because in the experimental setup human participants were randomly assigned to the adversary and defender roles. The *d* and σ parameters were calibrated in all the three experimental conditions using the human data collected in these conditions. In these calibrations, we minimized the sum of root mean square deviations (RMSDs) on attack and defend actions between model and human data. The RMSD was defined as:

(4) $$R\,M\,S\,D=\sqrt{\frac{1}{50}\,\sum_{t=1}^{50}{\left(m\,o\,d\,e\,l_{t}-h\,u\,m\,a\,n_{t}\right)}^{2}}$$

Where *model*_*t*_ and *human*_*t*_ refer to the average proportion of actions in trial *t* from model and human participants, respectively, and *t* refers to the trial number from 1 to 50. The RMSD has units of a proportion similar to the attack or defend action proportion. Smaller the value of RMSD, the better is the model’s fit to human data. Genetic algorithm (GA; Holland, 1992), which is an optimization algorithm, was used to calibrate *d* and σ parameters for both model participants. GA is known to generate high-quality solutions to optimization problems by relying on bio-inspired operators such as mutation, crossover, and selection (Mitchell, 1998). In the GA, the *d* and σ parameters were varied between 0.0 and 30.0. These ranges ensured that the optimization could capture the optimal parameter values with high confidence. In the GA, the crossover and mutation rates were kept at their default values of 80% and 1%, respectively. The GA’s stopping criteria was when there was no change in the fitness function for the last 50 generations. There were 50 parameter tuples per agent in each generation of the GA.

In the ACT-R model, we set *d* = 0.5 and σ = 0.25, i.e., the default values of the *d* and *s* parameters. The smaller values of *d* (∼ 0.5) and *s* parameters (∼ 0.25) indicate lesser reliance on recency and frequency and smaller variability in trial-to-trial decisions, respectively. Also, higher values of *d* parameter indicate a greater reliance on recency and frequency of outcomes and higher values of σ parameter indicate an increased trial-to-trial variability in decisions. We compared the performance of the calibrated model and ACT-R model in explaining human data.

Table 1 shows the parameters and RMSDs obtained for both the adversary and defender model players across the three conditions. As seen in Table 1, the RMSDs for the adversary and defender roles were small, suggesting a good model fit to human data (RMSDs <=20%, i.e., a deviation of less than or equal to 20% between model and human decisions can be considered as a good model fit in literature in this area (Aggarwal, 2018). Across all conditions, the calibrated *d* values in the IBL model were much higher compared to its default ACT-R value (=0.5) for both adversaries and defenders. Also, the calibrated *σ* parameter values were much higher compared to the ACT-R value (=0.25) for both adversaries and defenders. Thus, both adversaries and defenders showed excessive reliance on recency and frequency of experienced outcomes as well as greater trial-to-trial variability in decisions in the calibrated model.

**TABLE 1:** Parameter and RMSDs from the models across EQP, PDF, and PDM conditions.

Condition	Model	d_A_	d_D_	σ_A_	σ_D_	RMSD_A_	RMSD_D_
EQP	Calibrated-IBL	27.67	27.67	9.10	9.10	0.15	0.11
	ACT-R	0.50	0.50	0.25	0.25	0.18	0.15
PDF	Calibrated-IBL	28.41	28.41	13.20	13.20	0.18	0.15
	ACT-R	0.50	0.50	0.25	0.25	0.30	0.2
PDM	Calibrated-IBL	29.57	29.57	8.43	8.43	0.13	0.12
	ACT-R	0.50	0.50	0.25	0.25	0.16	0.31

*The subscript “D” and “A” refer to the defender and adversary roles; respectively.*

### IBL Model Generalization

**FIGURE 5:**
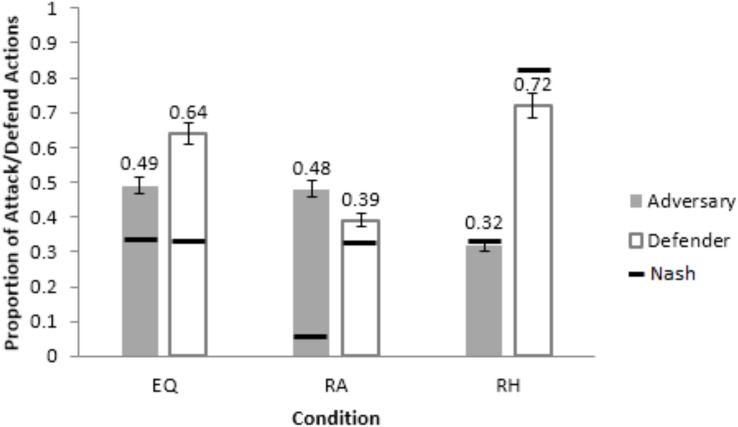
Average proportion of attack and defend actions from adversaries and defenders across the three conditions, Equal-Payoff (EQ),_:_ Rewarding Analyst (RA) and Rewarding Hacker (RH). The horizontal bars show the corresponding optimal/Nash proportions. The error-bars show the 95% CI around the mean.

The RMSDs obtained by the ACT-R model were smaller compared to the calibrated model in the calibration conditions for both adversary and defender roles (see Table 1). Thus, next, we evaluated the performance of the calibrated and ACT-R models in a neutral setting by generalizing both models to human data in Maqbool et al. (2017). Maqbool et al. (2017) made participants perform as hackers and analysts across three between-subject conditions: Equal payoff (EQ), where the payoffs were same as the EQP condition in the current study; rewarding hacker (RH), where the adversaries were rewarded 10-times the Equal Payoff for an undetected attack; and, rewarding analyst (RA), where the defenders were rewarded 10-times the Equal Payoff for a successful defense. In each condition, half of the participants were human defenders playing against Nash adversaries (adversary bots playing as per Nash proportion of attack actions) and half were human adversaries playing against Nash defenders (defenders bots playing as per Nash proportion of defend actions). In each condition, the calibrated and ACT-R models replaced the human player and played against the Nash player. The conditions used and the results obtained in the current experiment are different from those in Maqbool et al. (2017), except for those in the EQP condition. The current experiment’s results in EQP condition (Figure 5) agree with Maqbool et al. (2017)’s results in the EQ condition (Figure 5, where defenders defended excessively (64%) for a smaller proportion of Nash attack actions (33%).

Table 2 shows the RMSDs obtained during the generalization of calibrated and ACT-R model parameters from the current experiment to different conditions in Maqbool et al. (2017). For this generalization, all set of calibrated parameters from Table 1 were generalized to all conditions in Maqbool et al. (2017). For example, the PDF condition’s parameters from Table 1 were generalized to the EQ, RA, and RH conditions in the Maqbool et al. (2017) (see the second row in Table 2). As shown in Table 2, we found that the PDF condition’s parameters in the calibrated model generalized best to the EQ condition for the adversary role (RMSD = 0.19). The PDM condition’s parameters in the calibrated model generalized best to the EQ condition for the defender role (RMSD = 0.13). Similarly, it can be observed that the PDF condition’s parameters in the calibrated model generalized best to the RA condition for adversary role (RMSD = 0.16). The EQP condition’s parameters in the calibrated model generalized best in the RA condition for defender (RMSD = 0.15). Furthermore, the EQP condition’s parameters in the calibrated model generalized best in the RH condition for the adversary role (RMSD = 0.16). The PDM condition’s parameters in the calibrated model generalized best in the RH condition for the defender role (RMSD = 0.15). Also, we found the calibrated model to perform better compared to the ACT-R model across all cases of best generalization of parameters to Maqbool et al. (2017) conditions.

**TABLE 2:** The Generalization of IBL model and its parameters to different conditions in Maqbool et al. (2017).

		Generalization conditions					
Calibration condition	Model	EQ (Maqbool et al., 2017)		RA (Maqbool et al., 2017)		RH (Maqbool et al., 2017)	
		RMSD_A_	RMSD_D_	RMSD_A_	RMSD_D_	RMSD_A_	RMSD_D_
EQP	Calibrated IBL	0.26	0.25	0.28	**0.15**	**0.16**	0.30
	ACT-R	0.22	0.29	0.25	0.12	0.18	0.41
PDF	Calibrated IBL	**0.19**	0.48	**0.16**	0.23	0.24	0.54
	ACT-R	0.29	0.40	0.24	0.2	0.44	0.46
PDM	Calibrated IBL	0.35	**0.13**	0.34	0.26	0.20	**0.15**
	ACT-R	0.35	0.31	0.33	0.16	0.20	0.33

*The subscript “D” and “A” refer to the defender and adversary roles, respectively. Boldfaced values correspond to minimum RMSD values.*

**FIGURE 6:**
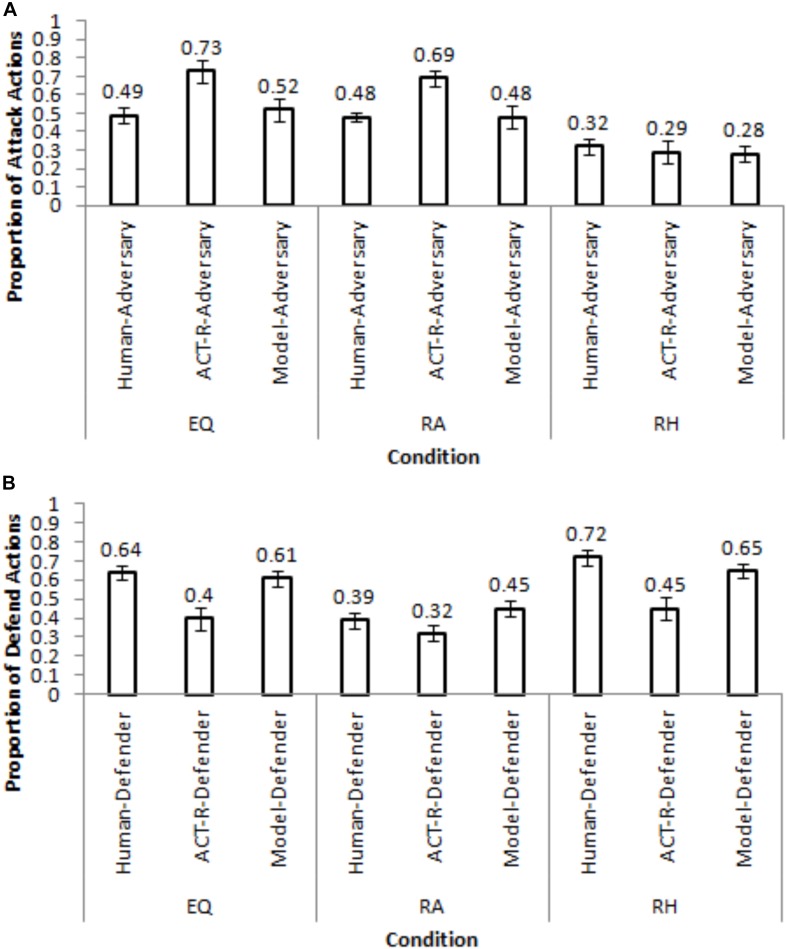
The generalization of the best performing parameters in the calibrated model to different conditions in Maqbool et al. (2017) for the adversary role **(A)** and defender role **(B)**.

Figure 6 shows the calibrated model’s performance in different conditions of Maqbool et al. (2017) for a set of generalization parameters that yielded the lowest RMSDs in Table 2. In Figure 6, the PDF condition’s and PDM condition’s parameters generalized best (lowest RMSDs) in the EQ condition for the adversary and defender roles, respectively. The PDF condition’s and EQP condition’s parameters generalized best in the RA condition for the adversary and defender roles, respectively. Finally, the EQP condition’s and PDM condition’s parameters generalized best in the RH condition for the adversary and defender roles, respectively. Mostly, the calibrated model generalized reasonably well to the Maqbool et al. (2017) dataset with small RMSDs (<=20%).

## Discussion and Conclusion

In this paper, we investigated how monetary penalties on defenders for false-alarms and misses influenced the decision-making of participants performing as adversaries and defenders. Also, we used (IBLT; Dutt et al., 2013) to model the decisions of participants performing as adversaries and defenders across different conditions involving penalties and rewards. Results revealed that monetary penalties on defenders for false-alarms and misses influenced the defend proportions as well as the attack proportions. Furthermore, penalizing defender decisions made adversaries perform differently from Nash proportions. Defenders agreed with Nash proportions when penalized for false-alarms and deviated from the Nash proportions when penalized for misses. The parameters of the IBL model calibrated to defender’s and adversary’s actions indicated excessive reliance on recency, frequency, and variability processes among participants in the current study as well as those in Maqbool et al. (2017).

First, the defend proportions decreased and increased when defenders were penalized for false-alarms and misses, respectively. This result could be explained based upon IBLT. When a player is penalized for an action, then, according to IBLT, the perceived value (blended value) for the penalized action becomes less than that for other non-penalized action (Dutt et al., 2013). According to Dutt et al. (2013), in such a situation, the action that provides a higher blended value will be the one performed a higher number of times. Thus, when defenders were penalized for false-alarms, they decreased the defend proportions and moved toward the Nash proportions. However, when defenders were penalized for misses, they increased the defend proportions and deviated from their Nash proportions.

Second, attack proportions were also influenced by the monetary penalties on defenders for false-alarms and misses. Most likely the adversary’s perception of defender’s actions caused him to change his own actions. From IBLT, the blended values of attack and not-attack actions were influenced indirectly by the positive and negative payoffs due reduced or increased defend actions, respectively. This explanation is plausible because the adversary possessed information about different penalties on their defender opponents in the experiment and experiencing these payoffs in the game made adversaries to change their actions.

Third, the calibration of the IBL model to human data indicated excessive reliance on recency, frequency, and variability mechanisms among both adversary and defender participants. Due to the high decay (*d*) value, both adversaries and defenders relied excessively on recently and frequently occurring outcome information in the IBL model. This reliance on recency and frequency likely caused both adversaries and defenders to deviate significantly from Nash proportions in a majority of conditions. These deviations of attack and defend proportions from the Nash proportions are similar to the sluggish-beta movements in signal-detection theory (Wickens and Hollands, 2000).

Furthermore, we observed a high trial-to-trial variability among defenders and adversaries across all calibration conditions. One likely reason for this observation may be that penalizing defenders for false alarms and misses caused both defenders and adversaries to change their decisions based upon their opponent’s decisions. However, the trial-to-trial variability found in participant’s decisions could also be due to the availability of information about opponents’ trial-to-trial actions and payoff matrices. Overall, one needs to systematically evaluate the reasons for the presence of excessive variability in player’s decisions as part of future research.

In our model results, we also found the calibrated model to perform better compared to the model with ACT-R default parameters. One likely reason for this finding could be that in the experiment participants tended to rely heavily on recency, frequency, and variability mechanisms in making decisions across different conditions. When the defender was punished for her misses, ACT-R model for the adversary performed better compared to the ACT-R model for the defender. Although we can only speculate, this difference in the MSDs between adversary and defender ACT-R models could be because the smaller recency and cognitive noise values seem to fit the adversary’s actions better compared to those of defender’s actions.

We performed a laboratory experiment using abstract games, and our conclusions should be seen with these assumptions in mind. Yet, the results of this experiment have certain real-world implications. First, penalizing defenders for their misses may not be a good strategy as it caused them to overshoot their optimal Nash proportions. However, penalizing defenders for misses also caused adversaries to deviate from their Nash proportions. Thus, organizations, who take the risk of penalizing defenders for misses, may also reap the rewards by making adversaries perform non-optimally. Furthermore, under the assumptions of the game played in the experiment, penalizing defenders for false-alarms could be a better organizational strategy compared to penalizing them for misses. That is because penalizing defenders for false-alarms made defenders to perform as per their Nash proportions and made adversaries to deviate from their Nash proportions. Overall, the deviations from Nash proportions for both players suggest that both players possessed cognitive limitations on memory and recall (Dutt et al., 2013; Maqbool et al., 2017) and did not perform as rational agents in the game (Wickens and Hollands, 2000).

Although we utilized concepts from behavioral game theory as well as cognitive science to investigate how monetary penalties on defenders influenced decision-making of participants performing as adversaries and defenders, the external and ecological validity of this lab-based study needs to be discussed. For example, in the real-world, cyber-defense exercises may involve high fidelity and the experimental paradigm discussed here may be an abstraction of the real-world phenomena. Similarly, under real-world conditions, defenders may work in teams, i.e., decisions to protect systems are likely made in groups. In this study, we investigated the decision-making of defenders as single individuals. Although this investigation is important, future research may extend the single individual game-theoretic paradigm to multiple individuals. Although we could only speculate, we expect that group decisions to be more informed compared to single-individual decisions, where cognitive biases and interests shown by certain individuals in the group maybe moderated by other individuals in the group. From the IBL model perspective, it would be interesting to investigate individual models interacting in the group, where the preferences of different individual models may be combined using different aggregation assumptions. Here, models that average the opinions of different bounded-rational agents with filtering of certain opinions (Shang, 2018b) may prove to be useful.

Furthermore, in our experiment, defenders and adversaries knew their actions but did not know whether these actions will succeed when they made decisions. In our setup, if a defender defended and the adversary attacked, then it was 100% guaranteed that the defender’s action would succeed. This scenario matches well with the real world, where the defenders, after installing an anti-virus, maybe 99.99% confident that it is likely to succeed. However, it may be worthwhile to contextualize the defend actions as successful for certain kinds of attacks and not successful for other kinds of attacks as part of future research. Still, beyond monetary motivations, several other factors like information availability among opponents and technology constraints (ability of a network to respond to defender’s actions) are likely to influence adversary’s and defender’s decisions in cyber-security games. We plan to investigate some of these ideas as part of our research in the near future.

## Data Availability Statement

The datasets generated for this study are available on request to the corresponding authors.

## Ethics Statement

The studies involving human participants were reviewed and approved by the Indian Institute of Technology Mandi. The patients/participants provided their written informed consent to participate in this study.

## Author Contributions

PA contributed to the design of the study, the implementation of experimental protocols, and data collection. ZM contributed to the data analyses and development of models, and wrote the manuscript. VD and VP developed the idea of the study, and contributed to the design, implementation of the study, and writing of the manuscript.

## Conflict of Interest

The authors declare that the research was conducted in the absence of any commercial or financial relationships that could be construed as a potential conflict of interest.
